# Characterization of B cells in healthy pregnant women from late pregnancy to post-partum: a prospective observational study

**DOI:** 10.1186/s12884-016-0927-7

**Published:** 2016-06-06

**Authors:** Jorge Lima, Catarina Martins, Maria J. Leandro, Glória Nunes, Maria-José Sousa, Jorge C. Branco, Luís-Miguel Borrego

**Affiliations:** Department of Obstetrics and Gynecology, CUF Descobertas Hospital, Lisbon, Portugal; CEDOC, Chronic Diseases Research Center, Immunology, NOVA Medical School, Faculty of Medical Sciences, Lisbon, Portugal; Center for Rheumatology Research, Department of Medicine, University College London, London, UK; Centro de Medicina Laboratorial Germano Sousa, Lisbon, Portugal; Department of Clinical Pathology, Hospital Prof. Fernando Fonseca, E.P.E., Amadora, Portugal; Obstetrics and Gynecology, Lisbon, Portugal; Department of Immunoallergy, CUF Descobertas Hospital, Lisbon, Portugal

**Keywords:** B cell subsets, Flow cytometry, Human pregnancy, Obstetrics

## Abstract

**Background:**

B cells play a role in pregnancy due to their humoral and regulatory activities. To our knowledge, different maturational stages (from transitional to memory) of circulating B cell subsets have not yet been characterized (cell quantification and phenotype identification) in healthy pregnant women. Thus, the objective of our study was to characterize these subsets (as well as regulatory B cells) from late pregnancy to post-partum and to compare them with the circulating B cells of non-pregnant women.

**Methods:**

In all of the enrolled women, flow cytometry was used to characterize the circulating B cell subsets according to the expression of IgD and CD38 (Bm1-Bm5 classification system). Regulatory B cells were characterized based on the expression of surface antigens (CD24, CD27, and CD38) and the production of IL-10 after lipopolysaccharide stimulation.

**Results:**

Compared to the absolute counts of B cells in the non-pregnant women (*n* = 35), those in the pregnant women (*n* = 43) were significantly lower (*p* < 0.05) during the 3rd trimester of pregnancy and on delivery day (immediately after delivery). The percentages of these cells on delivery day and at post-partum were significantly lower than those in the non-pregnant women.

In general, the absolute counts and percentages of the majority of the B cell subsets were significantly lower in the 3rd trimester of pregnancy and on delivery day than in the non-pregnant women. However, these counts and percentages did not differ significantly between the post-partum and the non-pregnant women.

The most notable exceptions to the above were the percentages of naïve B cells (which were significantly higher in the 3rd trimester and on delivery day than in the non-pregnant women) and of CD24^hi^CD38^hi^ regulatory B cells (which were significantly higher in the post-partum than in the non-pregnant women).

**Conclusion:**

According to our study, the peripheral B cell compartment undergoes quantitative changes during normal late pregnancy and post-partum. Such findings may allow us to better understand immunomodulation during human pregnancy and provide evidence that could aid in the development of new strategies to diagnose and treat pregnancy-associated disturbances. Our findings could also be useful for studies of the mechanisms of maternal responses to vaccination and infection.

**Electronic supplementary material:**

The online version of this article (doi:10.1186/s12884-016-0927-7) contains supplementary material, which is available to authorized users.

## Background

The immune system of pregnant women tolerates a genetically foreign fetus. This physiological adaptation is thought to be promoted by an array of anti-inflammatory and pro-inflammatory cytokines that are produced by T and B cells [1]. This state of immunological tolerance may influence the course of pre-existing pathologies (e.g., autoimmune diseases) [2] and may also increase fetal-maternal susceptibility to infection [3]. Furthermore, a decrease of function of B cells with loss of responsiveness to mitogens and infectious agents during the course of normal human pregnancy has also been reported [4].

B cells have a role in pregnancy because of their humoral activity (i.e., the production of protective antibodies against paternal antigens during pregnancy and the production of auto-antibodies that may lead to pregnancy complications) [5]. Normally, B cells leave the bone marrow and enter the circulation as immature transitional B cells, which later mature into naïve B cells. Finally, when naïve B cells encounter their cognate antigens in secondary lymphoid organs, these cells become activated and mature into memory B cells and plasma cells [6, 7]. B cell subsets of different maturational stages, from transitional to memory B cells, have been identified in peripheral blood using the mature B (Bm)1-Bm5 classification system. This classification system has proven to be effective in the identification of disturbances in the proportions of peripheral blood B cell subsets in patients with autoimmune diseases (e.g., Lupus or Sjögren’s syndrome) [8–10] and in those undergoing therapy (e.g., with biological agents), by assessing the depletion and repopulation of B cells [11].

Furthermore, it has been suggested that, in addition to their humoral activity, specific B cells can also have a regulatory function although this is still controversial. According to recent studies, regulatory B cells (Bregs) can inhibit pro-inflammatory responses by secreting the anti-inflammatory cytokine IL-10 [12, 13]. Breg counts increase in the first trimester of pregnancy, suppressing unwanted immune responses of maternal effector T cells, protecting against pregnancy loss [14]. While the phenotype and function of regulatory T cells has been extensively studied [15], further studies are needed to investigate the mechanisms behind the activation and expansion of Bregs and other B cell subsets in pregnancy.

These regulatory functions have been attributed to different B cell subsets, and despite some controversy, great progress has been made in the characterization of Bregs. The inability to identify a Breg-specific transcription factor, together with the phenotypic heterogeneity of Bregs, supports the idea that Bregs are not lineage specific and that they may expand in response to inflammation when immunosuppression is necessary [16]. It remains unclear whether the regulatory B cell function is a specific role of a particular subset or whether it is a reflection of their maturation stage. Although the expression of IL-10 has been a valuable tool in defining populations of Bregs, CD24^hi^CD27^+^ and CD24^hi^CD38^hi^ are the most frequently characterized phenotypes in humans [9, 12, 13].

Several studies [17–27] both prospective and cross-sectional have reported on circulating B cells during normal human pregnancy with majority describing lower total numbers and/or frequency when compared to levels post-partum or in healthy non-pregnant. Most studies [17, 19–24, 27] have only investigated total B cells as defined by the expression of CD20 or CD19 with older studies [18, 25, 26] using either expression of Ia (HLA-DR) or surface immunoglobulin. A few studies [17, 19, 24, 27] have reported on the frequency of B cell subsets expressing CD5 with majority describing lower frequency or lower total numbers of this subset during pregnancy, at delivery or early in the postpartum period. One study [19] reported lower CD21 and CD23 frequencies at delivery. However, peripheral B cells have not been characterized in human pregnancy while considering the different maturational stages, from transitional to memory B cells (using CD38 and immunoglobulin IgD as differentiation markers). Consequently, the objective of our study was to characterize these specific peripheral blood B cell subsets (transitional, naïve, unswitched memory, post-germinal, and resting memory B cells as well as plasmablasts) and Bregs (CD24^hi^CD27^+^, CD24^hi^CD38^hi^ and IL-10 regulatory B cells) from late pregnancy to post-partum and compare them with those in non-pregnant women.

## Methods

### Study population

This prospective observational study followed healthy pregnant women over time to characterize (i.e., cell quantification and phenotype identification) their peripheral blood B cell subsets from late pregnancy to post-partum. This characterization of B cells in the pregnant women was also compared with the characterization of single samples of peripheral blood B cells from a control group of healthy non-pregnant women to investigate changes associated with pregnancy.

Sequential non-laboring healthy women with singleton pregnancies who were attending an outpatient clinic (routine obstetrical care) during the 3rd trimester were recruited for participation. None of the pregnancies had complications prior to recruitment. Furthermore, all of the fetuses exhibited appropriate growth (as measured by uterine fundal height and by ultrasound performed after 28 weeks of gestation).

Sequential non-pregnant women who were attending an outpatient clinic were also recruited (healthy controls). These were asymptomatic women who were attending their annual routine well-woman exams.

For all of the women, the exclusion criteria were a history of diabetes, hypertension, or autoimmune disease and smoking during the 6 months prior to peripheral blood sample collection. Additional exclusion criteria for the pregnant women included prenatal use of any medication (other than vitamins and iron supplements) and ongoing complications in the pregnancy. Non-pregnant women taking oral contraceptives were also excluded, as these drugs affect circulating B cells [28].

All of the women were recruited at the *Hospital CUF Descobertas* in Lisbon (Portugal) between July 2013 and March 2014. The Ethics Committee of this hospital approved the study protocol. All of the recruited women provided written informed consent before the start of the study.

### Study visit procedures

Three visits were planned for the pregnant women: visit 1 was planned for the 3rd trimester of pregnancy (3rd trimester); visit 2, for the day of delivery; and visit 3, for post-partum (at least 6 weeks after delivery). A single visit was planned for the non-pregnant controls.

To characterize B cell subsets from late pregnancy to post-partum, peripheral blood samples were collected from all of the pregnant women at each planned visit: the “3rd trimester” sample was collected at visit 1, the “on delivery day” sample was collected at visit 2 (immediately after delivery, within 15 min after placental expulsion and oxytocin administration), and the “post-partum” sample was collected at visit 3. A peripheral blood sample was collected from the non-pregnant women at the planned visit, which took place during the follicular phase of their menstrual cycle because hormone status during the luteal phase is similar to that during pregnancy [29].

The baseline data collected for all women at the time of enrollment included demographics (age and ethnicity), anthropometrics [body mass index (BMI)], obstetric history, and systolic and diastolic blood pressures. The data collected for the pregnant women on the day of delivery included gestational age, type of analgesia and/or anesthesia, and mode of delivery. The data collected for the newborns included gender, weight, and 1-min and 5-min Apgar scores.

### Flow cytometry analysis and laboratory measurements

Peripheral blood samples were collected into EDTA-coated and heparinized tubes. These samples were analyzed by four-color flow cytometry (BD FACSCalibur, BD Biosciences, San Jose, CA, USA) to characterize B cell subsets and their maturation profiles. MultisetTM and CellQuest 3.3TM (BD Biosciences) software were used for both acquisition and analysis.

To obtain absolute counts of B cells (CD19^+^), a single-platform strategy was used. EDTA samples were assayed using a lyse-no-wash technique, with a BD IMK Kit with BD Trucount™ Tubes (BD Biosciences). The assay was performed according to the manufacturer’s instructions. In brief, 50 μL of blood were incubated for 15 min in the dark, at room temperature, with the monoclonal antibodies provided in the kit, in Trucount™ tubes containing a calibrated number of microbeads for counting purposes. Red blood cells were then lysed with the lysing solution (also provided with the BD IMK Kit), for 15 min and finally samples were acquired. The cells were gated on CD45/SSC, and a minimum of 2500 lymphocyte events were acquired. Multiset software provided percentage and absolute counts of B cells using the number of microbeads in each Trucount™ tube, along with the number of microbead and lymphocyte events acquired in each tube.

**Fig. 1:**
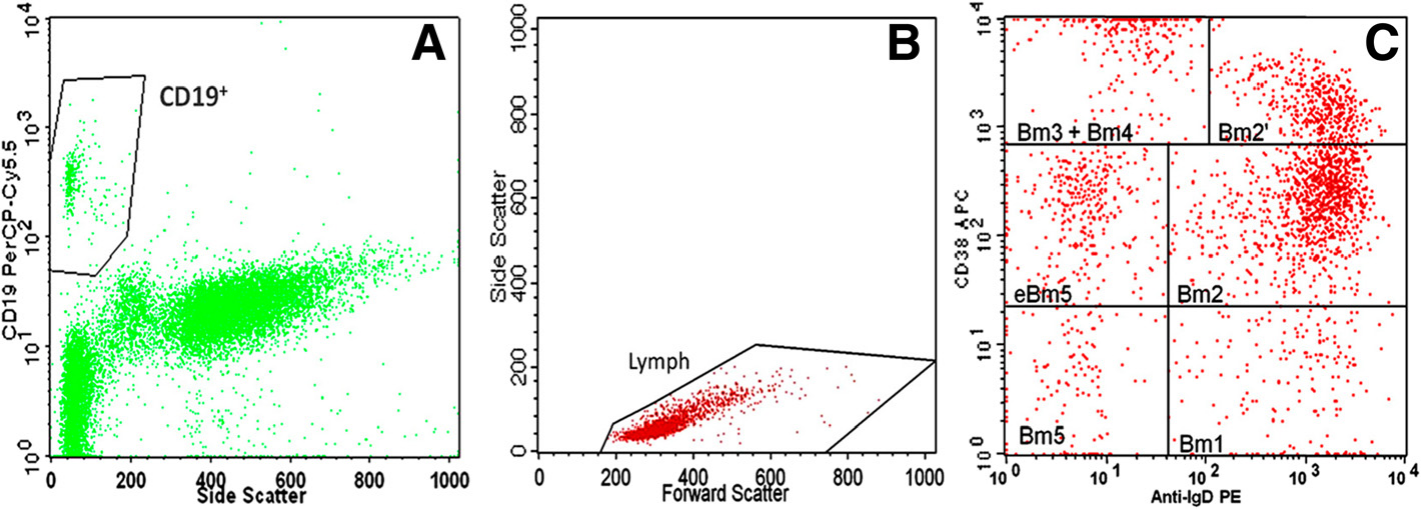
Identification of B cell subsets according to Bm1-5 classification system. **a** and **b** Gating strategy for CD19^+^ B cells using an initial CD19/SSC plot and refinement of the gate using a plot of FSC vs SSC. **c** Bm1-5 classification from double staining for IgD and CD38 (unswitched memory Bm1: IgD^+^CD38^−^; naïve Bm2: IgD^+^CD38^+^; transitional Bm2′: IgD^+^CD38^hi^; plasmablasts Bm3 + Bm4: IgD^−^CD38^hi^; post-germinal memory/early eBm5: IgD^−^CD38^+^; and resting memory/late Bm5: IgD^−^CD38^−^)

**Fig. 2:**
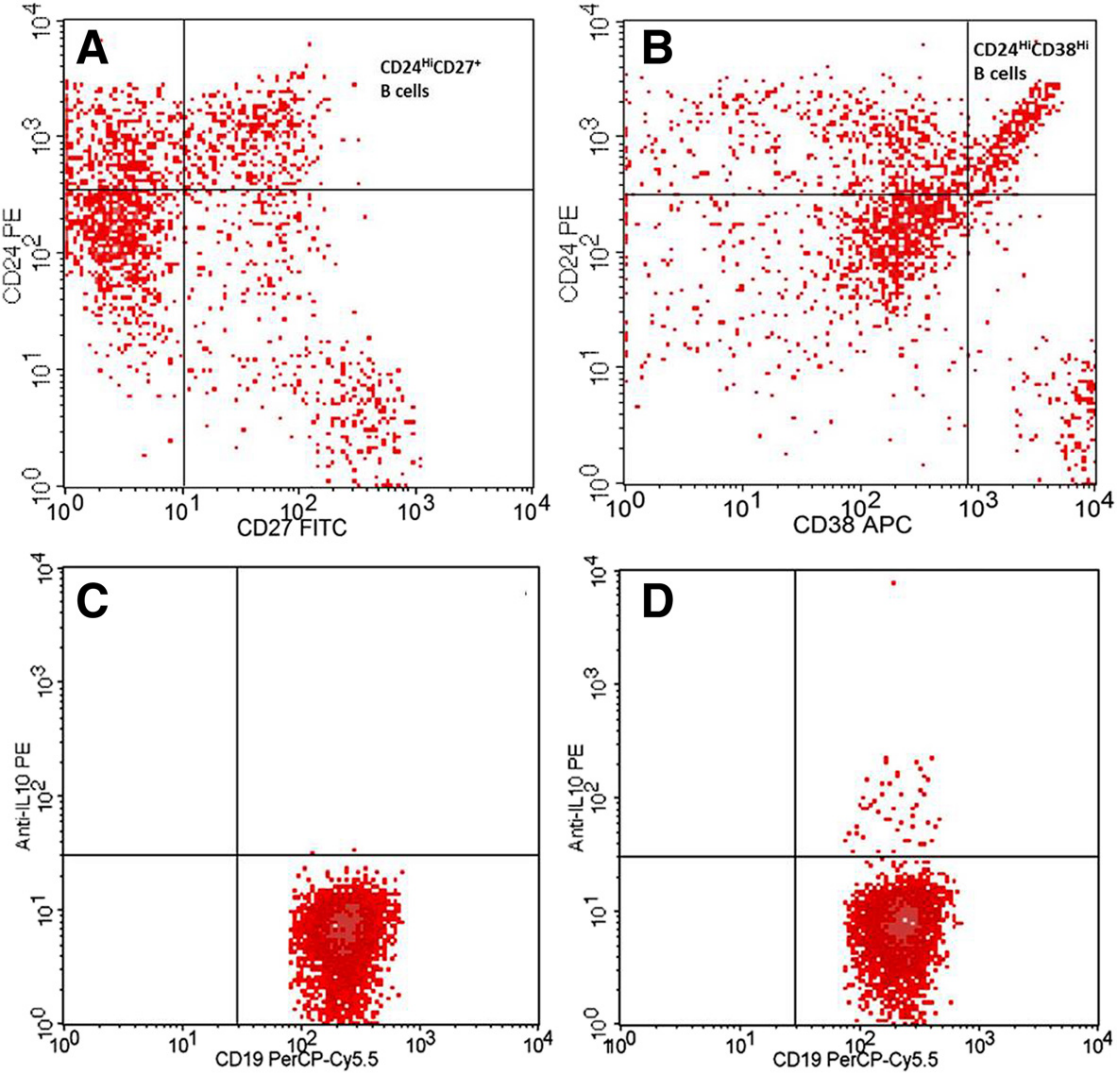
Identification of regulatory B cell subsets. **a** and **b** Gating strategy for CD24^hi^CD27^+^ (**a**) and CD24^hi^CD38^hi^ (**b**) Bregs; **c** and **d** IL-10-producing CD19^+^ B cells (CD19^+^ B cells were analyzed for the expression of IL-10 after a 5-h incubation period without stimulation (**c**) and with stimulation (**d**) with phorbol 12-myristate 13-acetate, calcium ionophore and lipopolysaccharide)

To study the surface B cell markers, a modified lyse-wash protocol was used. EDTA samples were washed twice in phosphate-buffered saline (PBS) to lower background staining. The washed cells were then stained with a panel of monoclonal antibodies (mAbs) that were conjugated with different fluorochromes: anti-CD19 PerCPCy5.5 (clone HIB19, Biolegend), anti-CD24 PE (clone ML5, Biolegend), anti-CD27 FITC (clone O323, Biolegend), CD38 APC (clone HIT2, Biolegend), and anti-IgD PE (clone IA6-2, BD Pharmingen). Red blood cells were incubated for 15 min at room temperature in the dark. The red cells were then lysed with BD FACS lysing solution (BD Biosciences) according to the manufacturer’s instructions. After a wash step with PBS, events were acquired. For the characterization of IL-10-producing Bregs, heparin samples were incubated for 5 h at 37 °C in a 5 % CO_2_ atmosphere with phorbol 12-myristate 13-acetate (PMA) (50 ng/mL, Sigma Aldrich), calcium ionophore (1 μg/mL, Sigma Aldrich), and lipopolysaccharide (LPS) (10 μg/mL, Sigma Aldrich) in the presence of Brefeldin A (1.0 μg/ml, BD Pharmingen) [13, 30]. After the stimulation, the red blood cells were lysed via the addition of BD FACS lysing solution and were stained for surface markers with anti-CD3 FITC (clone SK7, BD Biosciences), anti-CD19 PerCPCy5.5 (clone HIB19, Biolegend), and anti-CD8 APC (clone SK1, Biolegend) mAbs. The Cytofix-Cytoperm kit (BD Pharmingen) was used for cell fixation and permeabilization according to the manufacturer’s instructions. To assess the cytoplasmic expression of IL-10 in the B cells, a final intracellular staining step with an anti-IL-10 PE mAb (clone JES3-19F1, Biolegend) was performed before cell acquisition. A minimum of 2000 B cells (CD19+) were acquired in all tubes (gate in CD19/SSC). The analysis strategies are presented in Figs. 1 and 2. The flow cytometry results are presented as a percentage of total B cells and as absolute cell counts (cells/μL).

The Bm1-5 classification system used to identify the development of mature B cells was based on the expression of IgD/CD38 phenotypic markers. The cells were characterized as follows: transitional B cells (Bm2’: IgD^+^CD38^hi^), naïve B cells (Bm2: IgD^+^CD38^+^), unswitched memory B cells (Bm1: IgD^+^CD38^−^), and switched memory B cells (Bm5: IgD^−^CD38^+/−^) and were subsequently divided into post-germinal memory B cells (early Bm5: IgD^−^CD38^+^), resting memory (late Bm5: IgD^−^CD38^−^) B cells, and plasmablasts (Bm3 + Bm4: IgD^−^CD38^hi^) [8–10, 14]. Bregs were evaluated in three different populations: CD24^hi^CD27^+^, CD24^hi^CD38^hi^ and IL-10-producing B cells.

Our laboratory measurement included both absolute counts and percentages of total B cells and the different B cell subsets, as we feel that the two types of data are complementary. Percentages were measured as these allow interpreting the relative fluctuations in distinct B cell subsets from pregnancy to post-partum. Absolute counts were also measured and reported, although we are aware that pregnancy is characterized by variable degrees of hemodilution, and that changes in these counts may not reflect true variations in the total numbers of circulating cells.

### Statistical analysis

If the baseline data were normally distributed, they were presented as means (±standard deviations); otherwise, these data were presented as medians and ranges. Categorical variables were described as absolute and relative frequencies and were expressed as percentages.

Cell counts and percentages were presented as medians and ranges. If normally distributed, 2 independent groups were compared using Student’s t-tests; otherwise, Mann–Whitney U tests were used. If normally distributed, pairs of samples were compared using paired Student’s t-tests; otherwise, Wilcoxon signed-rank tests were used. For normally distributed data, comparisons between more than 2 groups were performed using ANOVA I; otherwise, Kruskal-Wallis tests were used. Statistical significance was defined by a *P*-value <0.05. The *P*-values for the comparisons of B cells between the non-pregnant women and pregnant women at different visits, as well as for the comparisons of the B cells of the pregnant women between visits, were adjusted for multiplicity using the Benjamini and Yekutieli method [31]. All of the data were analyzed using R software, version 3.12 for Windows.

## Results

### Baseline characteristics

A total of 78 women were enrolled in the study (43 pregnant and 35 non-pregnant). The characteristics of these women and of their newborns are presented in Table 1. The mean BMI of the non-pregnant women was 21.5 (±2.8) Kg/m^2^, while for the pregnant, it was 26.2 (±2.8) Kg/m^2^. All of the women were normotensive [mean systolic blood pressure for the non-pregnant was 119.8 (±10.5) mmHg, while for the pregnant, it was 115.7 (±9.3) mmHg; mean diastolic blood pressure for the non-pregnant was 74.7 (±7.4) mmHg, while for the pregnant, it was 67.4 (±7.4) mmHg]. Among the non-pregnant women, the median number of weeks since the last pregnancy (regardless of whether the pregnancies were interrupted or resulted in a live birth) was 169 (23–449). The median gestational age in the 3rd trimester of pregnancy was 33.0 (31–35) weeks, while it was 39.0 (37–41) weeks on the day of delivery. The pregnant group was significantly younger (*p* = 0.016) and included significantly more nulliparous women (*p* < 0.001) than the non-pregnant group. All of the pregnant women, regardless of the mode of delivery, received regional analgesia and/or anesthesia. No general anesthesia was administered to these women. All of the pregnant women were discharged from the hospital 2 days after a vaginal delivery or 3 days after a cesarean section. Final post-partum measurements were carried out a median of 45 (41–58) days after delivery.

**Table 1:** Characteristics of the women enrolled in the study and of their newborns

	Non-pregnant women (*n* = 35)	Pregnant women (*n* = 43)
Age in years, median (range)	35.0 (20–40)	32.0 (25–41)*
Ethnicity, *n* (%)		
White	35 (100)	42 (97.8)
Black	0	1 (2.2)
Gestational age in weeks, median (range)		
3rd trimester		33.0 (31–35)
Day of delivery		39.0 (37–41)
Parity, *n* (%)		
Nulliparous	5 (14.3)	24 (55.8)*
Primiparous	14 (40)	18 (41.9)
Multiparous	16 (45.7)	1 (2.3)
Mode of delivery, *n* (%)		
Vaginal		18 (41.8)
Cesarean		25 (58.2)
Elective cesarean^a^		14 (55.6)
Intrapartum cesarean^b^		11 (44.4)
Newborns		
Birth weight in grams, mean (± SD)		3265.0 (±393.5)
Gender, *n* (%)		
Male		22 (51)
Female		21 (49)
APGAR score, median (range)		
1-min Apgar score		9 (6; 10)
5-min Apgar score		10 (9; 10)
5-min Apgar score less than 7		0 (0)

Note: *SD* standard deviation; ^a^preformed prelabor; ^b^performed in labor; *statistically significant differences (*p* < 0.05) between pregnant and non-pregnant

### Characterization of the B cell population (CD19^+^)

**Fig. 3:**
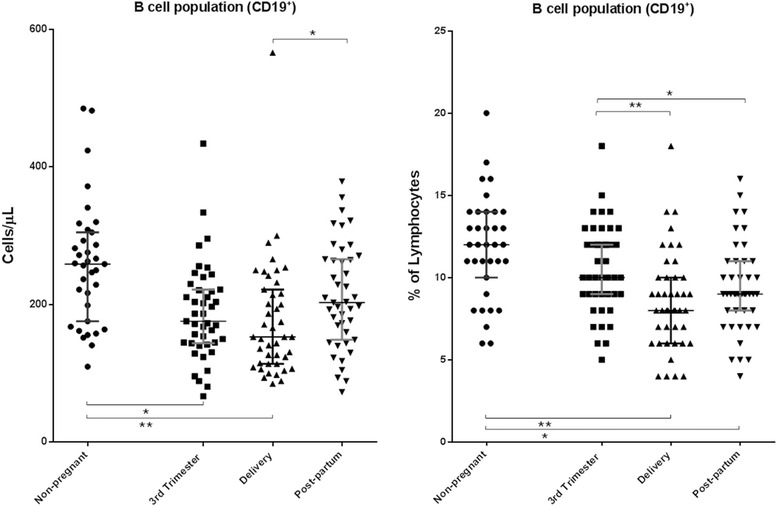
B cell population in peripheral blood samples. Delivery, within 15 min after placental expulsion; Post-partum, at least 6 weeks after delivery; The *bottom line* represents the 25th percentile, the *top line* represents the 75th percentile, and the *middle line* represents the median. * *p* < 0.05; ** *p* < 0.001

The characterization of the B cell population for all of the enrolled women is presented in Fig. 3. The median absolute numbers (259 [110–485]) and percentages (12 [6–20]) of these cells in the non-pregnant were within the expected normal ranges of our protocol (absolute count: 80–616 cells/μL; percentage: 5–22 %). The absolute counts of B cells at delivery were significantly lower (*p* < 0.05) than those at post-partum and in the non-pregnant women. Furthermore, the absolute counts of these cells during the 3rd trimester were also significantly lower (*p* < 0.05) than those in the non-pregnant. The percentages of B cells at delivery and at post-partum were significantly lower than those in the pregnant women during the 3rd trimester of pregnancy and in the non-pregnant.

### Characterization of maturational stages of B cells

**Fig. 4:**
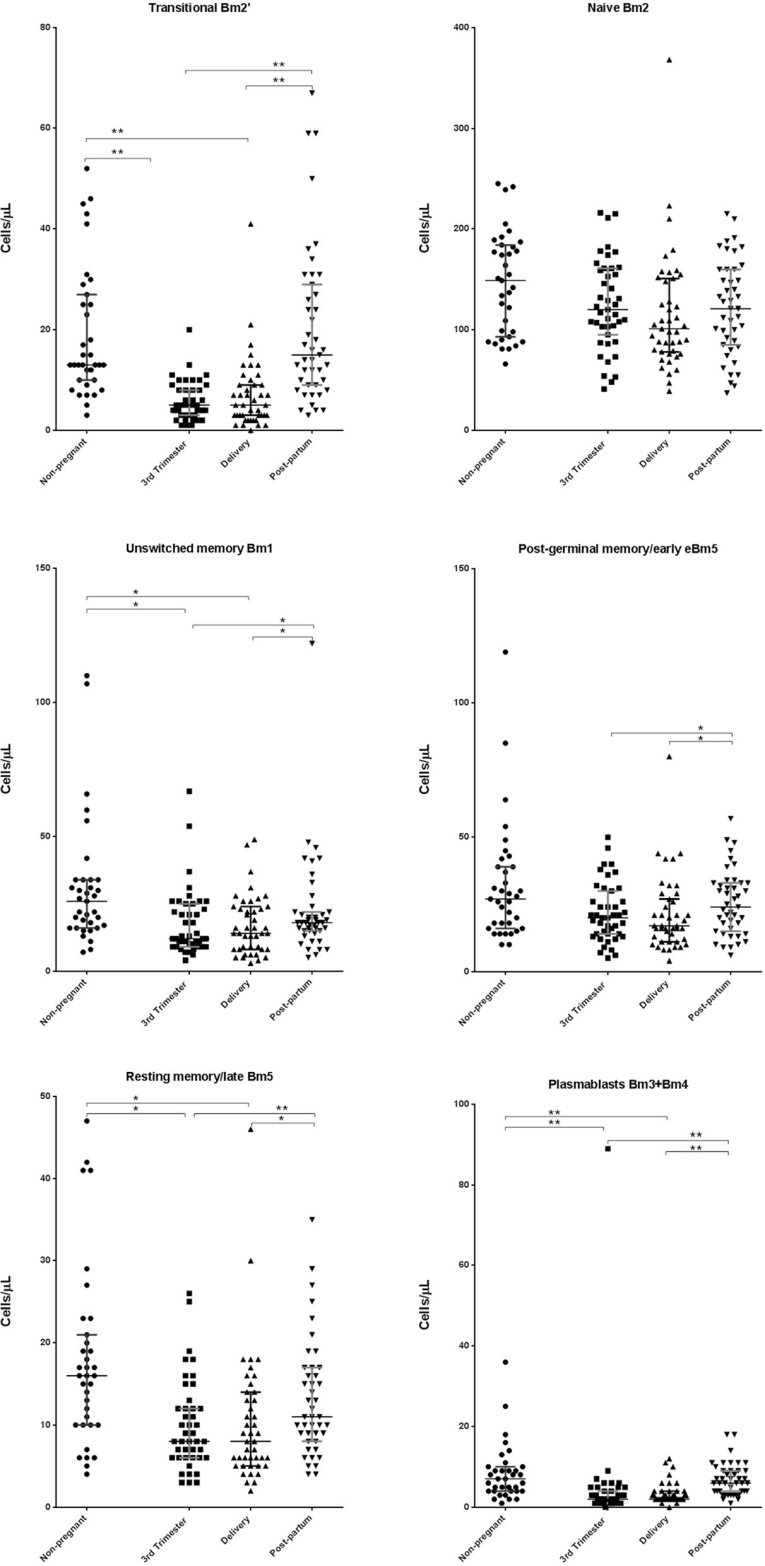
Maturational stages of B cells (absolute counts) in peripheral blood samples according to Bm1-5 classification. Bm1-5 classification, IgD/CD38 cell surface markers; Delivery, within 15 min after placental expulsion; Post-partum, at least 6 weeks after delivery; Non-pregnant women. The *bottom line* represents the 25th percentile, the *top line represents* the 75th percentile, and the *middle line* represents the median. * *p* < 0.05; ** *p* < 0.001

**Fig. 5:**
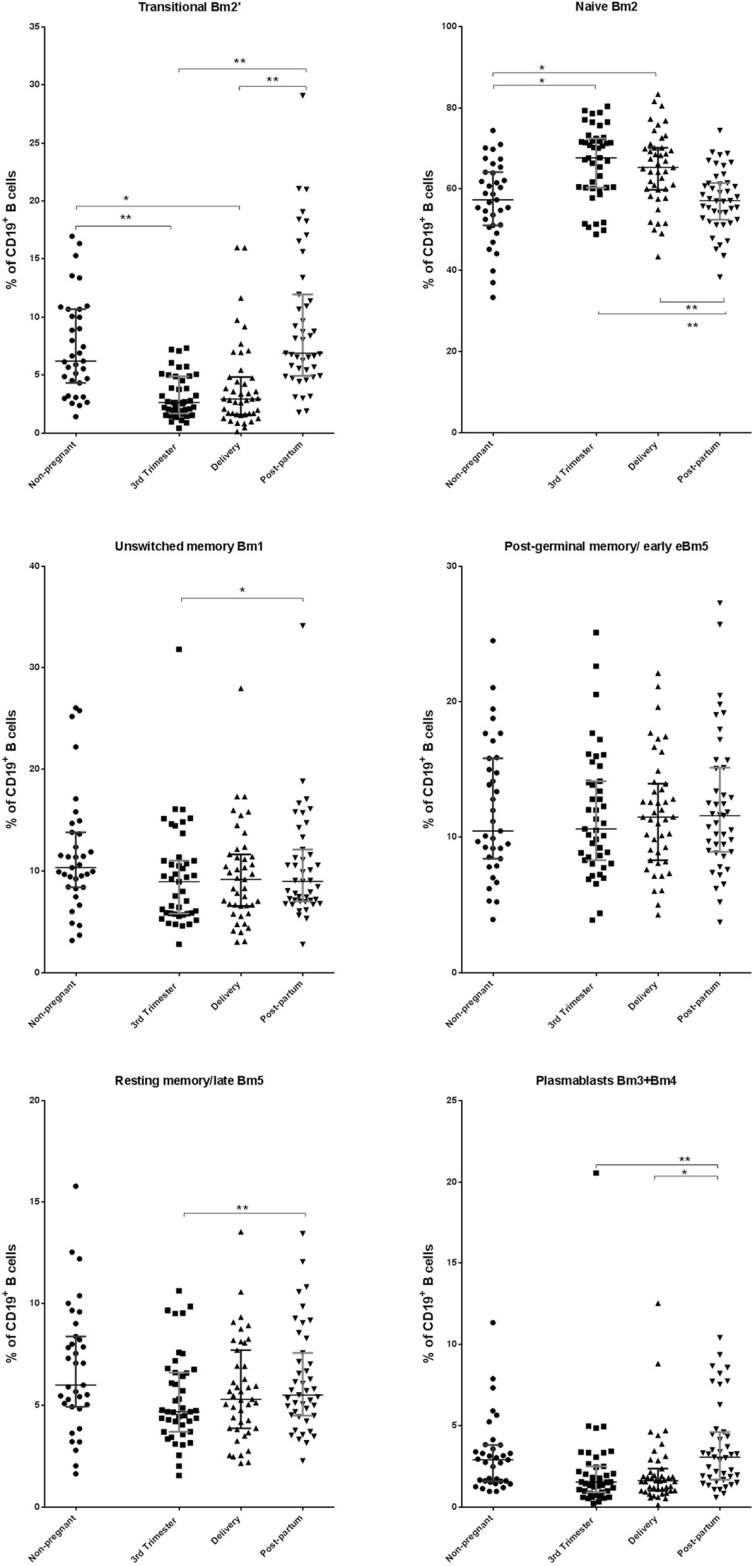
Maturational stages of B cells (percentages) in peripheral blood samples according to Bm1-5 classification. Bm1-5 classification, IgD/CD38 cell surface markers; Delivery, within 15 min after placental expulsion; Post-partum, at least 6 weeks after delivery; Non-pregnant women. The *bottom line* represents the 25th percentile, the *top line* represents the 75th percentile, and the *middle line* represents the median. * *p* < 0.05; ** *p* < 0.001

The characterization of specific B cell subsets (transitional, naïve, unswitched memory, post-germinal, and resting memory B cells as well as plasmablasts) for all of the enrolled women is presented in Fig. 4 (absolute counts) and Fig. 5 (percentages). The absolute counts of transitional B cells, unswitched memory B cells, resting memory B cells, and plasmablasts during the 3rd trimester of pregnancy and on delivery day were significantly lower (*p* < 0.05) than the corresponding counts in the non-pregnant. The absolute counts of naïve and of post-germinal memory/early B cells did not significantly differ (*p* ≥ 0.05) between the pregnant and non-pregnant women at any of the study visits. The absolute counts of all of the B cell subsets, excluding naïve B cells, were significantly higher (*p* < 0.05) at post-partum compared to those during the 3rd trimester of pregnancy and on delivery day.

The percentages of transitional B cells in the 3rd trimester of pregnancy and on delivery day were significantly lower (*p* < 0.05) than those in the non-pregnant and post-partum women. Conversely, the percentages of naïve B cells in the 3rd trimester of pregnancy and on delivery day were significantly higher (*p* < 0.05) compared those in both the non-pregnant and post-partum women. No significant differences (*p* < 0.05) in the percentages of unswitched memory, post-germinal memory, and resting memory B cells as well as of plasmablasts were identified between the pregnant and non-pregnant women at any of the study visits. However, the percentages of unswitched memory and resting memory B cells and plasmablasts were significantly lower (*p* < 0.05) during the 3rd trimester compared to post-partum. Furthermore, the percentages of plasmablasts were also significantly lower (*p* < 0.05) on delivery day compared to post-partum.

### Characterization of Breg

**Fig. 6:**
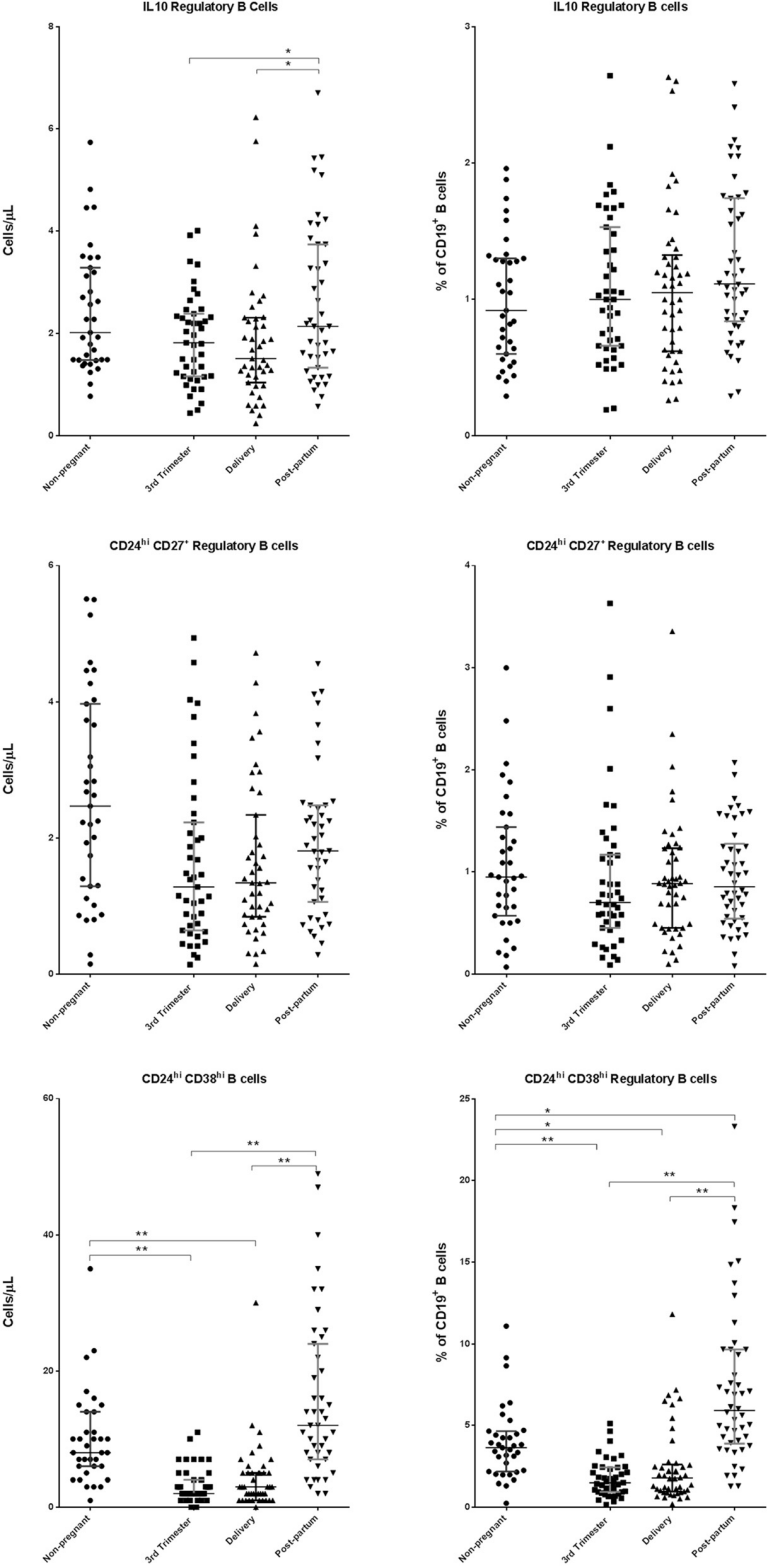
Regulatory B cells in peripheral blood samples (absolute counts and percentages). Delivery, within 15 min after placental expulsion; Post-partum, at least 6 weeks after delivery; Non-pregnant women. The *bottom line* represents the 25th percentile, the *top line* represents the 75th percentile, and the *middle line* represents the median. * *p* < 0.05; ** *p* < 0.001

The characterization of Breg is presented in Fig. 6. The absolute counts of IL-10 regulatory B cells and CD24^hi^CD38^hi^ Bregs during the 3rd trimester of pregnancy and on delivery day were significantly lower (*p* < 0.05) than those in the post-partum women. Additionally, the absolute counts of CD24^hi^CD38^hi^ Bregs were also significantly lower (*p* < 0.05) during the 3rd trimester of pregnancy and on delivery day compared to the corresponding counts in the non-pregnant women. The absolute counts of CD24^hi^CD27^+^ Bregs did not significantly differ between the pregnant and non-pregnant women or between study visits.

The percentages of CD24^hi^CD38^hi^ Bregs during the 3rd trimester of pregnancy and on delivery day were significantly lower (*p* < 0.05) than those in the non-pregnant and post-partum women. Furthermore, the percentages of these cells at post-partum were significantly higher (*p* < 0.05) than those in the non-pregnant women. The percentages of IL-10 regulatory B cells and of CD24^hi^CD27^+^ Bregs did not significantly differ between the pregnant and non-pregnant women or between study visits.

## Discussion

According to our study, late-stage pregnancy (between the 3rd trimester and delivery) is associated with peripheral blood B cell lymphopenia. Indeed, the absolute counts and percentages of most B cell subsets in the 3rd trimester of pregnancy and on delivery day were significantly lower compared to the corresponding counts and percentages in the non-pregnant. However, these differences did not significantly differ between the post-partum and non-pregnant women, suggesting that at this later time point, the absolute counts and percentages of most B cell subsets revert (or at least partially revert) to normal values. The most notable exceptions to this observation were identified for naïve B cells (whose percentages were significantly higher during the 3rd trimester and on delivery day than in the non-pregnant) and for CD24^hi^CD38^hi^ Bregs (whose percentages were significantly higher in the post-partum compared to the non-pregnant women).

To our knowledge, this is the first study to characterize the circulating B cell compartment in pregnancy while taking into account the maturational stages of the different B cell subsets. Furthermore, the study was conducted prospectively, from the 3rd trimester of pregnancy to post-partum, in a sample of 43 pregnant women.

The pregnancy-associated B lymphopenia that we identified in our study has already been described in animal models [32, 33] and in humans [17–27]. According to Medina et al. [32], B lymphopoiesis in bone marrow is selectively reduced during normal pregnancy because of hormonal influences. Furthermore, Muzzio et al. [33] demonstrated that B lymphopoiesis is reduced in late pregnancy, when estradiol levels are high. Cellular migration is another mechanism that can contribute to B cell lymphopenia. Studies of animal models have shown that monocytes and other immune cells migrate to the uterus during the later stages of pregnancy due to changes in the expression of chemokines [34, 35]. Furthermore, small populations of B cells have been identified in the decidua, suggesting leucocyte recruitment into the maternal-fetal interface [36]. The biological meaning of this suppression of B lymphopoiesis in normal pregnancy is uncertain but is probably related to the physiological immune tolerance.

In this study, we found that the absolute counts of the majority of the B cell subsets were significantly lower in the 3rd trimester of pregnancy than in the non-pregnant women, suggesting pregnancy-associated B lymphopenia (though most pregnant women still presented values within normal ranges). The increased blood volemia observed during pregnancy could in part explain these observations. In fact, pregnant presented also decreased absolute lymphocyte counts, along with decreased absolute counts of T and NK cells, compared to non-pregnant (data not shown). Nonetheless, B cell percentages in pregnant also seem to decrease during pregnancy, which does not happens with either T cells or NK cells.

Compared to the percentages of peripheral blood naïve B cells in the non-pregnant women, we found higher values during the 3rd trimester and on delivery day, but no differences were observed in absolute counts. This relative increase in naïve B cells may be a consequence of decreased differentiation of B cells into memory cells and/or plasmablasts. In fact, Muzzio et al. [33] reported the expansion of naïve B cells in pregnant mice. The high levels of progesterone present in late pregnancy may potentially explain this, as high progesterone levels inhibit B cell activation in mice [37]. Our results may also be explained by the mobilization of more differentiated B subsets from peripheral blood to other body tissues.

Normal pregnancy has been compared to a state of quiescent systemic inflammation, while parturition has been likened to an immunological reaction that results in the recruitment of immune cells not only to the maternal-fetal interface but also to the systemic circulation [38]. The results of our study support this idea, as we identified higher counts and percentages of CD24^hi^CD38^hi^ Bregs post-partum relative to during the 3rd trimester and on delivery day. This observation may represent a regulatory mechanism for the suppression of immune cell activation events and may also explain the increased susceptibility to infections that occurs during the post-partum period and the altered clinical outcomes of some autoimmune diseases.

Interestingly, we found that while the majority of B cell subsets increased to levels closer to those of the non-pregnant (or to normal values) from the third trimester of pregnancy to post-partum, the percentages of CD24^hi^CD38^hi^ Bregs were significantly higher in the post-partum compared to the non-pregnant women. Because B lymphopoiesis is under endocrine regulation during pregnancy [32, 33], our results may be explained by the decline of hormonal levels that typically occurs post-partum, which may have lead both to lymphopoiesis recovery and to B cell activation. As hypothesized by Medina et al. [32], this result may be of clinical utility to increase lymphocyte formation in transplanted patients and in those with immunodeficiencies.

Unlike for the CD24^hi^CD38^hi^ Bregs, no significant differences between the non-pregnant and the pregnant or from the 3rd trimester of pregnancy to post-partum were identified for IL-10 Bregs (cell percentages) or for CD24^hi^CD27^+^ Bregs (cell percentages and counts). This heterogeneity of Breg subsets has been reported in other studies with humans [39]. Furthermore, CD24^hi^CD27^+^ Bregs, an activated memory subset, are more mature than transitional CD24^hi^CD38^hi^ Bregs; thus, it is more likely for them to develop into antibody-producing cells that no longer possess a regulatory function [40].

The differences between pregnant and non-pregnant women identified for age, and parity are not likely to bias our results. In fact, among all of the women who were included in our study, counts and percentages of B cell subsets were not significantly associated with age, as demonstrated by the non-significant Spearman correlation coefficients between these variables (see Additional file 1). Furthermore, in the vast majority of cases, there were no statistically significant differences in counts and percentages of B cell subsets among women, despite parity (see Additional file 2). Finally, in the vast majority of cases, we have also not found significant associations between counts (and percentages) of B cell subsets and gestational age at the 3rd trimester of pregnancy (see Additional file 3), gestational age at delivery (see Additional file 4), length of time post-partum until the collection of the final blood samples (see Additional file 5), and time since last pregnancy in the non-pregnant (see Additional file 6).

Previous studies have identified differences in the counts and percentages of B cells (total and subsets) between neonates and individuals of up to 50 years of age [41]. However, to the best of our knowledge, there are no data regarding B cell variation in women over short periods of time, such as that of our study.

Although the pregnant women received analgesia and/or anesthesia, which may cause temporary changes in maternal blood pressure, this is unlikely to cause important changes in B cell counts because regional administration is generally associated with low plasma levels of these drugs. Ideally, samples collected before pregnancy would have been compared with samples collected during pregnancy in the same individuals; however, this would have been very difficult for us from a practical point of view. The fact that several of the changes that were observed during the 3rd trimester of pregnancy seem to be reversed during the post-partum period suggests that comparisons with non-pregnant women were adequate.

In future research, it is important to investigate whether B cell subset characterization could help to identify risk markers for the development of obstetric complications in pregnant women with or without autoimmune diseases. In this context, it would also be important to clarify the role of B-cell activating factor (BAFF), an essential survival factor for transitional B cells, and of CD23, a B-lymphocyte differentiation marker.

## Conclusion

According to our study, the characteristics of peripheral B cell compartment differ significantly between pregnant and non-pregnant women and vary over time from late pregnancy to post-partum. Such findings may allow us to recognize normal fluctuations in B cell subsets to better understand immune regulation during human pregnancy and to identify new strategies for the diagnosis and treatment of pregnancy-associated disturbances as well as the mechanisms of maternal responses to vaccination and infection.

## Abbreviations

Bm, mature B cells. Bregs, regulatory B cells.

## Additional files

Additional file 1: Correlation coefficients between counts (and percentages) of B cell subsets and age, both for pregnant and non-pregnant women. ^a^Healthy non-pregnant women; ^b^Third trimester of pregnancy; ^c^Within 15 min after placental expulsion; ^d^At least 6 weeks after delivery; ^e^Spearman correlation coefficient. (XLSX 11 kb) Additional file 2: Comparison of counts (and percentages) of B cell subsets between nulliparous, primiparous, and multiparous women, both for pregnant and non-pregnant women. Values presented as median (Interquartile range); Three classes were compared using one-way ANOVA, or Kuskal-Wallis tests; Two classes were compared with t-Student or Wilcoxon tests; There was only one multiparous women among the pregnant, and this woman was excluded from this analysis. ^a^Non-pregnant women; ^b^Third trimester of pregnancy; ^c^Within 15 min after placental expulsion; ^d^At least 6 weeks after delivery; ^e^Kuskal-Wallis test; f Wilcoxon test; * *p* < 0.05. (XLSX 46 kb) Additional file 3: Correlation coefficients between counts (and percentages) of B cell subsets and gestational age at the 3rd trimester of pregnancy. Corr. Coef., Spearman correlation coefficient. (XLSX 9 kb) Additional file 4: Correlation coefficients between counts (and percentages) of B cell subsets and gestational age at delivery. Corr. Coef., Spearman correlation coefficient. (XLSX 9 kb) Additional file 5: Correlation coefficients between counts (and percentages) of B cell subsets and length of time post-partum until the collection of the final blood samples. Corr. Coef., Spearman correlation coefficient. (XLSX 9 kb) Additional file 6: Correlation coefficients between counts (and percentages) of B cell subsets and time since last pregnancy, in the non-pregnant women. Corr. Coef., Spearman correlation coefficient. (XLSX 9 kb)
